# Bis[2,6-bis­(1-methyl-1*H*-benzimidazol-2-yl-κ*N* ^3^)pyridine-κ*N*]zinc dipicrate methanol disolvate

**DOI:** 10.1107/S1600536812031443

**Published:** 2012-07-14

**Authors:** Xuyang Fan, Jingkun Yuan, Ying Bai, Jin Kong, Huilu Wu

**Affiliations:** a School of Chemical and Biological Engineering, Lanzhou Jiaotong University, Lanzhou 730070, People’s Republic of China

## Abstract

In the title compound, [Zn(C_21_H_17_N_5_)_2_](C_6_H_2_N_3_O_7_)_2_·2CH_3_OH, the Zn^II^ atom is coordinated by six N atoms from two tridentate 2,6-bis­(1-methyl-1*H*-benzimidazol-2-yl)pyridine ligands in a distorted octa­hedral environment. In the crystal, the picrate anions and methanol solvent mol­ecules are connected by O—H⋯O hydrogen bonds. Weak inter­molecular C—H⋯O hydrogen bonds are also observed.

## Related literature

For the applications of benzimidazole derivatives, see: Horton *et al.* (2003 ▶); Wang *et al.* (1994 ▶); Cowan (1998 ▶); Liu *et al.* (2004 ▶, 2011 ▶); Wright (1951 ▶). For a related crystal structure, see: Huang *et al.* (2010 ▶).
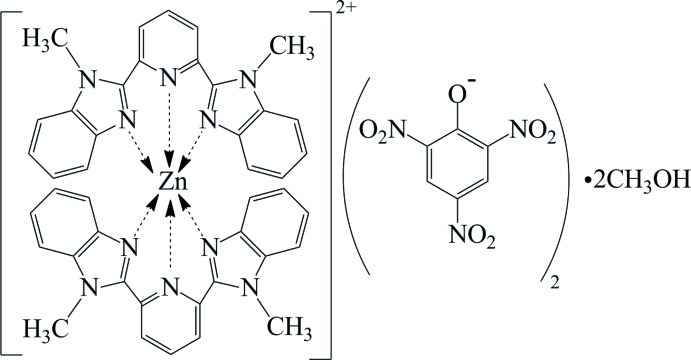


## Experimental

### 

#### Crystal data

[Zn(C_21_H_17_N_5_)_2_](C_6_H_2_N_3_O_7_)_2_·2CH_4_O
*M* *_r_* = 1264.46
Triclinic, 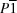

*a* = 13.2007 (3) Å
*b* = 13.8024 (3) Å
*c* = 16.2009 (3) Å
α = 80.811 (1)°
β = 71.012 (1)°
γ = 88.538 (1)°
*V* = 2754.28 (10) Å^3^
*Z* = 2
Mo *K*α radiation
μ = 0.54 mm^−1^
*T* = 153 K
0.38 × 0.36 × 0.30 mm

#### Data collection

Bruker APEXII area-detector diffractometer
Absorption correction: multi-scan (*SADABS*; Sheldrick, 1996 ▶) *T*_min_ = 0.816, *T*_max_ = 0.852
22669 measured reflections
10148 independent reflections
9024 reflections with *I* > 2σ(*I*)
*R*_int_ = 0.017

#### Refinement

*R*[*F* ^2^ > 2σ(*F* ^2^)] = 0.039
*wR*(*F* ^2^) = 0.111
*S* = 1.07
10148 reflections
811 parameters
H-atom parameters constrained
Δρ_max_ = 0.78 e Å^−3^
Δρ_min_ = −0.51 e Å^−3^

### 

Data collection: *APEX2* (Bruker, 2007 ▶); cell refinement: *SAINT* (Bruker, 2007 ▶); data reduction: *SAINT*; program(s) used to solve structure: *SHELXS97* (Sheldrick, 2008 ▶); program(s) used to refine structure: *SHELXL97* (Sheldrick, 2008 ▶); molecular graphics: *SHELXTL* (Sheldrick, 2008 ▶); software used to prepare material for publication: *SHELXTL*.

## Supplementary Material

Crystal structure: contains datablock(s) global, I. DOI: 10.1107/S1600536812031443/lh5492sup1.cif

Structure factors: contains datablock(s) I. DOI: 10.1107/S1600536812031443/lh5492Isup2.hkl

Additional supplementary materials:  crystallographic information; 3D view; checkCIF report

## Figures and Tables

**Table 1:** Hydrogen-bond geometry (Å, °)

*D*—H⋯*A*	*D*—H	H⋯*A*	*D*⋯*A*	*D*—H⋯*A*
O15—H15⋯O1^i^	0.84	1.94	2.756 (4)	165
C31—H31*A*⋯O2^i^	0.95	2.53	3.204 (3)	128
C42—H42*A*⋯O1^i^	0.98	2.32	3.106 (3)	137
C55—H55*C*⋯O9^ii^	0.98	2.56	3.415 (7)	146
C11—H11*A*⋯O11^iii^	0.95	2.39	3.207 (3)	144
C10—H10*A*⋯O3^iv^	0.95	2.37	3.233 (3)	152
C4—H4*A*⋯O4^v^	0.95	2.50	3.333 (3)	146
C37—H37*A*⋯O11^vi^	0.95	2.49	3.314 (3)	146
C10—H10*A*⋯O10^vii^	0.95	2.57	3.133 (4)	118
C20—H20*B*⋯O14^vii^	0.98	2.42	2.949 (3)	113

Symmetry codes: (i) 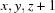; (ii) 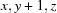; (iii) 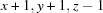; (iv) 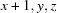; (v) 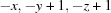; (vi) 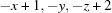; (vii) 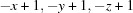.

**Fig. 1.**
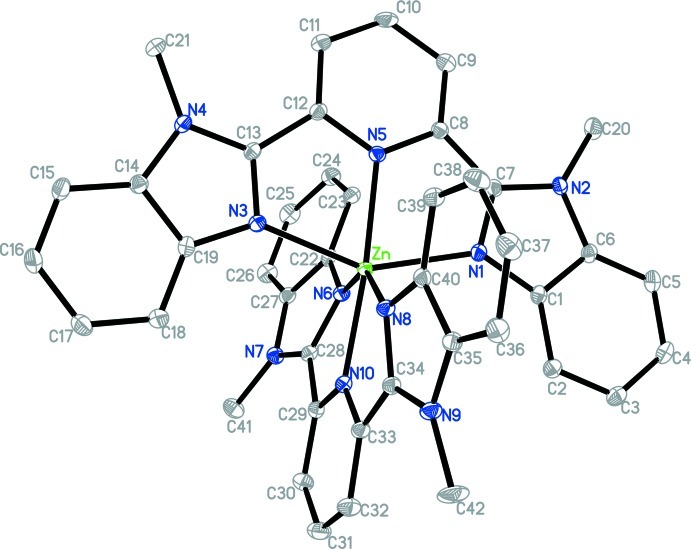
The molecular structure of the [ZnII(bmbp)2] cation. Displacement ellipsoids are drawn at the 30% probability level. H atoms and methanol solvate molecules have been omitted for clarity.

**Fig. 2.**
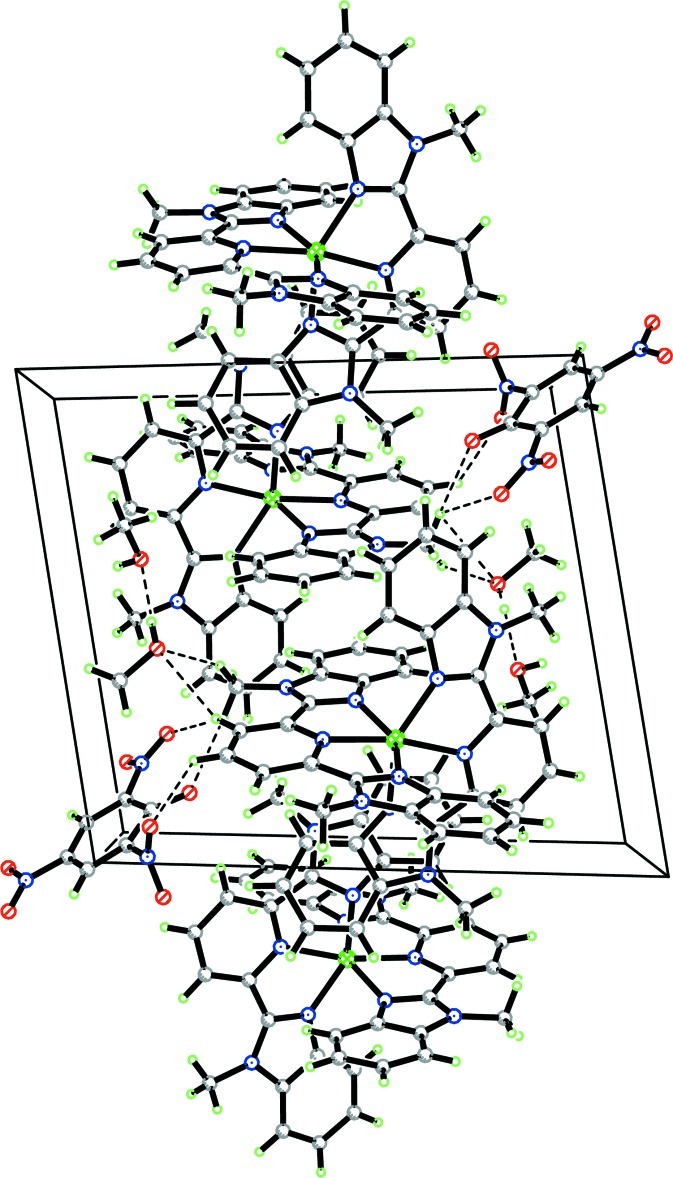
The crystal packing of the title compound. Intermolecular hydrogen bonds are shown as dashed lines.

## supplementary crystallographic information

### Comment

Benzimidazole and its derivatives have attracted considerable interests in recent years for their versatile properties in chemistry and pharmacology (Wang *et al.* 1994; Horton *et al.* 2003). As a part of the chemical structure of vitamin B_12_ (Wright 1951), benzimidazole scaffolds have shown wide application in medicine, fungicide, biochemical reagents and many other fields (Cowan 1998; Liu *et al.* 2004). Moreover, as a typical heterocyclic ligand, the large benzimidazole rings not only can provide potential supermolecule recognition sites for π···π stacking interactions, but also act as hydrogen bond acceptors and donors to assemble multiple coordination geometries (Liu *et al.* 2011). As part of our reasearch in this area we have already determined the crystal structure of bis[2,6-bis(1H-benzimidazol-2-yl)pyridine]nickel(II) dipicrate dimethylformamide disolvate (Huang *et al.*, 2010) and the crystal structure of the title compound is presented herein.

The asymmetric unit of the title complex consists of a [Zn^II^(bmbp)_2_] cation (bmbp = 2,6-bis(*N*-methylbenzimidazol-2-yl)pyridine) (Fig. 1), two picrate anions and two methanol solvent molecules. The Zn^II^ ion is coordinated by six N atoms from two two tridentate V-shaped ligands (bmbp) ligands in a distorted octacahedral enviroment. In the crystal, the picrate anions and solvent methanol molecules are connected by O—H···O hydrogen bonds. Weak intermolecular C—H···O hydrogen bonds are also observed (Fig. 2).

### Experimental

To a stirred solution of 2,6-bis(*N*-ethylbenzimidazol-2-yl)pyridine (0.1697 g, 0.50 mmol) in hot MeOH (10 ml) was added Zn(picrate)_2_ (0.1304 g, 0.25 mmol) solution dissolved in MeOH (5 ml). A yellow crystalline product formed rapidly immediately. The sediment was filtered off, washed with MeOH and absolute Et_2_O, and dried in *vacuo*. The crude product was dissolved in mixed MeOH-DMF solution to form a pale yellow solution into which Et_2_O was allowed to diffuse at room temperature. Yellow crystals of it suitable for X-ray measurement were obtained after two weeks. (found: C, 53.45; H, 3.84; N, 17.55. Calcd. for C_56_ H_46_ N_16_ O_16_ Zn: C, 53.27; H, 3.67; N, 17.75)

### Refinement

All H atoms were found in difference electron maps and were subsequently included in a riding-model approximation with C—H distances ranging from 0.95 to 0.98 Å and *U*_iso_(H) = 1.2 *U*_eq_(C),*U*_iso_(H) = 1.5*U*_eq_(C_methyl_).

### Crystal data

**Table:**

[Zn(C_21_H_17_N_5_)_2_](C_6_H_2_N_3_O_7_)_2_·2CH_4_O	*Z* = 2
*M**_r_* = 1264.46	*F*(000) = 1304
Triclinic, *P*1	*D*_x_ = 1.525 Mg m^−^^3^
Hall symbol: -P 1	Mo *K*α radiation, λ = 0.71073 Å
*a* = 13.2007 (3) Å	Cell parameters from 10153 reflections
*b* = 13.8024 (3) Å	θ = 3.1–25.5°
*c* = 16.2009 (3) Å	µ = 0.54 mm^−^^1^
α = 80.811 (1)°	*T* = 153 K
β = 71.012 (1)°	Block, yellow
γ = 88.538 (1)°	0.38 × 0.36 × 0.30 mm
*V* = 2754.28 (10) Å^3^	

### Data collection

**Table:**

Bruker APEXII area-detector diffractometer	10148 independent reflections
Radiation source: fine-focus sealed tube	9024 reflections with *I* > 2σ(*I*)
Graphite monochromator	*R*_int_ = 0.017
ω scans	θ_max_ = 25.5°, θ_min_ = 3.1°
Absorption correction: multi-scan (*SADABS*; Sheldrick, 1996)	*h* = −15→15
*T*_min_ = 0.816, *T*_max_ = 0.852	*k* = −16→16
22669 measured reflections	*l* = −19→19

### Refinement

**Table:**

Refinement on *F*^2^	Secondary atom site location: difference Fourier map
Least-squares matrix: full	Hydrogen site location: inferred from neighbouring sites
*R*[*F*^2^ > 2σ(*F*^2^)] = 0.039	H-atom parameters constrained
*wR*(*F*^2^) = 0.111	*w* = 1/[σ^2^(*F*_o_^2^) + (0.0558*P*)^2^ + 2.8031*P*] where *P* = (*F*_o_^2^ + 2*F*_c_^2^)/3
*S* = 1.07	(Δ/σ)_max_ = 0.001
10148 reflections	Δρ_max_ = 0.78 e Å^−^^3^
811 parameters	Δρ_min_ = −0.51 e Å^−^^3^
0 restraints	Extinction correction: *SHELXL97* (Sheldrick, 2008), Fc^*^=kFc[1+0.001xFc^2^λ^3^/sin(2θ)]^-1/4^
Primary atom site location: structure-invariant direct methods	Extinction coefficient: 0.0020 (4)

### Special details

**Table:**

Geometry. All esds (except the esd in the dihedral angle between two l.s. planes) are estimated using the full covariance matrix. The cell esds are taken into account individually in the estimation of esds in distances, angles and torsion angles; correlations between esds in cell parameters are only used when they are defined by crystal symmetry. An approximate (isotropic) treatment of cell esds is used for estimating esds involving l.s. planes.
Refinement. Refinement of *F*^2^ against ALL reflections. The weighted *R*-factor *wR* and goodness of fit *S* are based on *F*^2^, conventional *R*-factors *R* are based on *F*, with *F* set to zero for negative *F*^2^. The threshold expression of *F*^2^ > σ(*F*^2^) is used only for calculating *R*-factors(gt) *etc*. and is not relevant to the choice of reflections for refinement. *R*-factors based on *F*^2^ are statistically about twice as large as those based on *F*, and *R*- factors based on ALL data will be even larger.

### Fractional atomic coordinates and isotropic or equivalent isotropic displacement parameters (Å^2^)

**Table:**

	*x*	*y*	*z*	*U*_iso_*/*U*_eq_	
Zn	0.699284 (19)	0.754092 (17)	0.578747 (15)	0.01513 (9)	
O1	0.28834 (17)	0.53363 (17)	−0.01056 (13)	0.0503 (6)	
O2	0.26536 (18)	0.71973 (17)	0.03078 (17)	0.0626 (7)	
O3	0.09610 (17)	0.74158 (14)	0.08634 (14)	0.0466 (5)	
O4	−0.06207 (15)	0.51349 (16)	0.35968 (13)	0.0431 (5)	
O5	−0.0202 (2)	0.36062 (17)	0.36496 (15)	0.0604 (7)	
O6	0.2169 (3)	0.25914 (18)	0.08881 (19)	0.1090 (15)	
O7	0.3604 (2)	0.3520 (2)	0.03785 (19)	0.0708 (8)	
O8	0.31607 (15)	0.12803 (13)	0.81934 (11)	0.0343 (4)	
O9	0.1328 (3)	−0.0774 (3)	0.8911 (2)	0.0944 (11)	
O10	0.0861 (3)	0.0692 (2)	0.8926 (3)	0.1292 (18)	
O11	0.19845 (16)	−0.12180 (14)	1.19446 (12)	0.0403 (5)	
O12	0.33643 (18)	−0.04076 (16)	1.19439 (13)	0.0471 (5)	
O13	0.53377 (15)	0.18178 (15)	0.94195 (13)	0.0418 (5)	
O14	0.4566 (3)	0.2465 (2)	0.85048 (19)	0.0943 (12)	
O15	0.2472 (3)	0.6163 (3)	0.8375 (2)	0.1042 (12)	
H15	0.2632	0.6013	0.8840	0.125*	
O16	0.8403 (3)	0.5680 (3)	0.1703 (2)	0.0917 (10)	
H16	0.8169	0.5134	0.1665	0.110*	
N1	0.61233 (14)	0.67598 (13)	0.51819 (11)	0.0174 (4)	
N2	0.58800 (15)	0.65098 (13)	0.39274 (11)	0.0176 (4)	
N3	0.84613 (14)	0.82283 (13)	0.57262 (11)	0.0179 (4)	
N4	1.01794 (14)	0.86493 (13)	0.49867 (12)	0.0192 (4)	
N5	0.80558 (14)	0.75208 (12)	0.44653 (11)	0.0161 (4)	
N6	0.63124 (14)	0.89742 (13)	0.54757 (11)	0.0174 (4)	
N7	0.58053 (15)	1.03783 (13)	0.59946 (12)	0.0193 (4)	
N8	0.69979 (14)	0.62875 (13)	0.67234 (11)	0.0168 (4)	
N9	0.60273 (15)	0.53039 (14)	0.79475 (12)	0.0204 (4)	
N10	0.59056 (14)	0.78586 (13)	0.70167 (12)	0.0174 (4)	
N11	0.17438 (18)	0.68927 (17)	0.07385 (14)	0.0344 (5)	
N12	−0.01124 (18)	0.44612 (18)	0.32607 (15)	0.0368 (5)	
N13	0.2646 (3)	0.33716 (19)	0.07852 (16)	0.0504 (7)	
N14	0.1388 (2)	0.0044 (2)	0.90879 (16)	0.0447 (6)	
N15	0.27282 (17)	−0.06106 (16)	1.15817 (14)	0.0315 (5)	
N16	0.46173 (18)	0.18250 (17)	0.90974 (14)	0.0367 (5)	
C1	0.50998 (17)	0.63813 (15)	0.53891 (14)	0.0170 (4)	
C2	0.42797 (18)	0.61677 (15)	0.62057 (14)	0.0203 (5)	
H2A	0.4392	0.6247	0.6742	0.024*	
C3	0.33074 (19)	0.58396 (16)	0.61995 (15)	0.0235 (5)	
H3A	0.2735	0.5698	0.6742	0.028*	
C4	0.31384 (19)	0.57089 (17)	0.54111 (16)	0.0257 (5)	
H4A	0.2453	0.5487	0.5435	0.031*	
C5	0.39438 (19)	0.58948 (16)	0.46001 (15)	0.0230 (5)	
H5A	0.3833	0.5798	0.4068	0.028*	
C6	0.49255 (18)	0.62310 (15)	0.46096 (14)	0.0185 (4)	
C7	0.65650 (17)	0.68154 (15)	0.43119 (14)	0.0169 (4)	
C8	0.76679 (17)	0.72233 (15)	0.38747 (14)	0.0176 (4)	
C9	0.82556 (19)	0.73526 (18)	0.29843 (15)	0.0252 (5)	
H9A	0.7984	0.7129	0.2570	0.030*	
C10	0.92543 (19)	0.78191 (18)	0.27147 (15)	0.0267 (5)	
H10A	0.9671	0.7923	0.2106	0.032*	
C11	0.96532 (18)	0.81369 (17)	0.33225 (15)	0.0227 (5)	
H11A	1.0336	0.8458	0.3140	0.027*	
C12	0.90227 (17)	0.79702 (15)	0.42037 (14)	0.0180 (4)	
C13	0.92473 (17)	0.82849 (15)	0.49604 (14)	0.0171 (4)	
C14	0.99804 (18)	0.88374 (16)	0.58402 (14)	0.0195 (4)	
C15	1.06430 (19)	0.92029 (17)	0.62419 (16)	0.0259 (5)	
H15A	1.1372	0.9386	0.5926	0.031*	
C16	1.0183 (2)	0.92849 (18)	0.71214 (16)	0.0279 (5)	
H16A	1.0609	0.9527	0.7421	0.034*	
C17	0.9104 (2)	0.90203 (18)	0.75876 (16)	0.0270 (5)	
H17A	0.8820	0.9084	0.8195	0.032*	
C18	0.84469 (19)	0.86709 (17)	0.71870 (15)	0.0239 (5)	
H18A	0.7714	0.8504	0.7502	0.029*	
C19	0.89030 (17)	0.85717 (16)	0.62963 (14)	0.0193 (4)	
C20	0.6048 (2)	0.64939 (18)	0.29902 (14)	0.0264 (5)	
H20B	0.6226	0.7160	0.2658	0.040*	
H20A	0.5392	0.6251	0.2924	0.040*	
H20C	0.6637	0.6060	0.2762	0.040*	
C21	1.12344 (18)	0.8786 (2)	0.43008 (16)	0.0292 (5)	
H21A	1.1191	0.9272	0.3802	0.044*	
H21B	1.1462	0.8160	0.4096	0.044*	
H21C	1.1755	0.9017	0.4545	0.044*	
C22	0.65157 (16)	0.97291 (15)	0.47651 (14)	0.0181 (4)	
C23	0.69229 (17)	0.97117 (17)	0.38526 (14)	0.0209 (5)	
H23A	0.7124	0.9115	0.3628	0.025*	
C24	0.70186 (18)	1.05972 (17)	0.32951 (15)	0.0243 (5)	
H24A	0.7277	1.0605	0.2674	0.029*	
C25	0.6746 (2)	1.14826 (18)	0.36181 (16)	0.0274 (5)	
H25A	0.6842	1.2078	0.3212	0.033*	
C26	0.63384 (19)	1.15113 (17)	0.45178 (16)	0.0253 (5)	
H26A	0.6154	1.2111	0.4740	0.030*	
C27	0.62140 (17)	1.06121 (16)	0.50801 (14)	0.0195 (4)	
C28	0.58735 (17)	0.93899 (16)	0.61897 (14)	0.0177 (4)	
C29	0.55061 (17)	0.87549 (16)	0.70641 (14)	0.0187 (4)	
C30	0.47846 (19)	0.89771 (17)	0.78492 (15)	0.0236 (5)	
H30A	0.4505	0.9616	0.7885	0.028*	
C31	0.4488 (2)	0.82381 (18)	0.85764 (16)	0.0279 (5)	
H31A	0.3995	0.8371	0.9119	0.033*	
C32	0.49012 (19)	0.73043 (18)	0.85238 (15)	0.0254 (5)	
H32A	0.4695	0.6795	0.9021	0.031*	
C33	0.56265 (17)	0.71397 (16)	0.77206 (14)	0.0190 (4)	
C34	0.62009 (17)	0.62350 (16)	0.74933 (14)	0.0180 (4)	
C35	0.67656 (18)	0.47211 (16)	0.74403 (14)	0.0196 (4)	
C36	0.6950 (2)	0.37193 (17)	0.75803 (15)	0.0249 (5)	
H36A	0.6540	0.3296	0.8099	0.030*	
C37	0.7761 (2)	0.33797 (17)	0.69232 (16)	0.0289 (5)	
H37A	0.7916	0.2701	0.6993	0.035*	
C38	0.8371 (2)	0.40011 (18)	0.61503 (16)	0.0273 (5)	
H38A	0.8924	0.3732	0.5715	0.033*	
C39	0.81817 (18)	0.49872 (17)	0.60134 (14)	0.0212 (5)	
H39A	0.8589	0.5405	0.5491	0.025*	
C40	0.73637 (17)	0.53509 (15)	0.66761 (14)	0.0175 (4)	
C41	0.5416 (2)	1.10995 (17)	0.65917 (16)	0.0267 (5)	
H41A	0.5683	1.0945	0.7095	0.040*	
H41B	0.4631	1.1077	0.6807	0.040*	
H41C	0.5675	1.1758	0.6273	0.040*	
C42	0.5218 (2)	0.49229 (19)	0.87971 (16)	0.0356 (6)	
H42A	0.4525	0.5211	0.8818	0.053*	
H42B	0.5433	0.5096	0.9281	0.053*	
H42C	0.5154	0.4207	0.8860	0.053*	
C43	0.2241 (2)	0.5146 (2)	0.06625 (17)	0.0326 (6)	
C44	0.15790 (19)	0.58601 (18)	0.11362 (16)	0.0276 (5)	
C45	0.08051 (19)	0.56413 (18)	0.19506 (16)	0.0264 (5)	
H45A	0.0381	0.6145	0.2218	0.032*	
C46	0.06500 (19)	0.46772 (19)	0.23775 (16)	0.0285 (5)	
C47	0.1249 (2)	0.3934 (2)	0.19830 (17)	0.0341 (6)	
H47A	0.1130	0.3271	0.2272	0.041*	
C48	0.2016 (2)	0.4171 (2)	0.11696 (17)	0.0356 (6)	
C49	0.30545 (19)	0.09229 (17)	0.89718 (14)	0.0229 (5)	
C50	0.2194 (2)	0.02170 (18)	0.94949 (15)	0.0264 (5)	
C51	0.2092 (2)	−0.02996 (17)	1.03094 (16)	0.0275 (5)	
H51A	0.1530	−0.0777	1.0596	0.033*	
C52	0.28341 (19)	−0.01071 (17)	1.07077 (15)	0.0252 (5)	
C53	0.36580 (19)	0.05843 (18)	1.02961 (15)	0.0258 (5)	
H53A	0.4151	0.0712	1.0585	0.031*	
C54	0.37602 (19)	0.10873 (17)	0.94652 (15)	0.0245 (5)	
C55	0.1576 (6)	0.6855 (4)	0.8528 (4)	0.121 (2)	
H55A	0.0967	0.6559	0.9030	0.145*	
H55B	0.1359	0.6997	0.7998	0.145*	
H55C	0.1809	0.7466	0.8656	0.145*	
C56	0.8505 (5)	0.6345 (4)	0.0916 (3)	0.0966 (17)	
H56A	0.7805	0.6415	0.0827	0.116*	
H56B	0.9016	0.6095	0.0412	0.116*	
H56C	0.8764	0.6986	0.0964	0.116*	

### Atomic displacement parameters (Å^2^)

**Table:**

	*U*^11^	*U*^22^	*U*^33^	*U*^12^	*U*^13^	*U*^23^
Zn	0.01501 (14)	0.01619 (14)	0.01242 (13)	−0.00262 (9)	−0.00208 (9)	−0.00181 (9)
O1	0.0440 (12)	0.0594 (14)	0.0294 (10)	0.0121 (10)	0.0103 (9)	−0.0040 (10)
O2	0.0407 (13)	0.0534 (14)	0.0658 (15)	−0.0173 (11)	0.0128 (11)	0.0094 (12)
O3	0.0482 (13)	0.0319 (10)	0.0438 (12)	0.0051 (9)	0.0047 (9)	−0.0029 (9)
O4	0.0291 (10)	0.0536 (13)	0.0337 (10)	−0.0006 (9)	0.0064 (8)	−0.0049 (9)
O5	0.0675 (16)	0.0447 (13)	0.0449 (13)	−0.0072 (11)	0.0056 (11)	0.0132 (11)
O6	0.157 (3)	0.0329 (14)	0.0711 (19)	0.0022 (17)	0.051 (2)	−0.0056 (13)
O7	0.0545 (16)	0.0847 (19)	0.0792 (18)	0.0369 (14)	−0.0203 (14)	−0.0387 (15)
O8	0.0442 (11)	0.0370 (10)	0.0197 (9)	−0.0088 (8)	−0.0109 (8)	0.0039 (8)
O9	0.117 (3)	0.100 (2)	0.091 (2)	−0.023 (2)	−0.055 (2)	−0.0398 (19)
O10	0.126 (3)	0.077 (2)	0.229 (5)	−0.032 (2)	−0.148 (3)	0.044 (3)
O11	0.0401 (11)	0.0403 (11)	0.0287 (9)	−0.0055 (9)	−0.0044 (8)	0.0151 (8)
O12	0.0518 (13)	0.0590 (13)	0.0316 (10)	−0.0074 (10)	−0.0238 (10)	0.0129 (9)
O13	0.0308 (10)	0.0539 (12)	0.0392 (11)	−0.0127 (9)	−0.0146 (9)	0.0057 (9)
O14	0.109 (2)	0.099 (2)	0.0811 (19)	−0.0802 (19)	−0.0744 (18)	0.0726 (17)
O15	0.139 (3)	0.128 (3)	0.0483 (17)	0.005 (3)	−0.0323 (19)	−0.0185 (19)
O16	0.095 (2)	0.107 (3)	0.081 (2)	0.011 (2)	−0.0284 (18)	−0.040 (2)
N1	0.0185 (9)	0.0163 (9)	0.0168 (9)	−0.0022 (7)	−0.0049 (7)	−0.0025 (7)
N2	0.0213 (9)	0.0164 (9)	0.0161 (9)	−0.0020 (7)	−0.0073 (7)	−0.0027 (7)
N3	0.0178 (9)	0.0184 (9)	0.0161 (9)	−0.0032 (7)	−0.0035 (7)	−0.0019 (7)
N4	0.0143 (9)	0.0208 (9)	0.0208 (9)	−0.0024 (7)	−0.0029 (7)	−0.0042 (7)
N5	0.0178 (9)	0.0158 (8)	0.0134 (8)	−0.0008 (7)	−0.0035 (7)	−0.0016 (7)
N6	0.0172 (9)	0.0176 (9)	0.0156 (9)	−0.0012 (7)	−0.0034 (7)	−0.0010 (7)
N7	0.0200 (9)	0.0180 (9)	0.0201 (9)	0.0025 (7)	−0.0063 (7)	−0.0043 (7)
N8	0.0165 (9)	0.0191 (9)	0.0134 (8)	−0.0018 (7)	−0.0037 (7)	−0.0009 (7)
N9	0.0215 (10)	0.0209 (9)	0.0148 (9)	−0.0030 (7)	−0.0020 (7)	0.0011 (7)
N10	0.0146 (9)	0.0197 (9)	0.0177 (9)	−0.0015 (7)	−0.0046 (7)	−0.0034 (7)
N11	0.0337 (13)	0.0350 (12)	0.0267 (11)	−0.0066 (10)	0.0010 (9)	−0.0041 (9)
N12	0.0263 (11)	0.0442 (14)	0.0326 (12)	−0.0065 (10)	−0.0027 (9)	0.0013 (11)
N13	0.075 (2)	0.0405 (15)	0.0276 (12)	0.0190 (14)	−0.0077 (13)	−0.0047 (11)
N14	0.0501 (15)	0.0502 (15)	0.0335 (12)	−0.0234 (13)	−0.0204 (11)	0.0123 (12)
N15	0.0323 (12)	0.0335 (12)	0.0225 (10)	0.0014 (9)	−0.0055 (9)	0.0064 (9)
N16	0.0355 (13)	0.0432 (13)	0.0281 (11)	−0.0151 (10)	−0.0117 (10)	0.0093 (10)
C1	0.0192 (11)	0.0114 (9)	0.0205 (10)	0.0000 (8)	−0.0072 (9)	−0.0010 (8)
C2	0.0236 (11)	0.0164 (10)	0.0195 (11)	−0.0010 (8)	−0.0061 (9)	−0.0002 (9)
C3	0.0220 (12)	0.0216 (11)	0.0234 (11)	−0.0027 (9)	−0.0043 (9)	0.0005 (9)
C4	0.0199 (12)	0.0246 (12)	0.0318 (13)	−0.0058 (9)	−0.0084 (10)	−0.0012 (10)
C5	0.0255 (12)	0.0206 (11)	0.0265 (12)	−0.0023 (9)	−0.0132 (10)	−0.0037 (9)
C6	0.0216 (11)	0.0143 (10)	0.0194 (11)	−0.0023 (8)	−0.0073 (9)	−0.0003 (8)
C7	0.0197 (11)	0.0141 (10)	0.0172 (10)	−0.0009 (8)	−0.0062 (8)	−0.0031 (8)
C8	0.0201 (11)	0.0150 (10)	0.0168 (10)	−0.0008 (8)	−0.0046 (8)	−0.0024 (8)
C9	0.0254 (12)	0.0305 (12)	0.0197 (11)	−0.0014 (10)	−0.0053 (9)	−0.0079 (10)
C10	0.0250 (12)	0.0346 (13)	0.0139 (10)	−0.0007 (10)	0.0024 (9)	−0.0034 (10)
C11	0.0189 (11)	0.0250 (11)	0.0198 (11)	−0.0025 (9)	−0.0011 (9)	−0.0018 (9)
C12	0.0163 (10)	0.0165 (10)	0.0204 (11)	0.0005 (8)	−0.0048 (8)	−0.0032 (8)
C13	0.0162 (10)	0.0155 (10)	0.0177 (10)	−0.0024 (8)	−0.0040 (8)	−0.0003 (8)
C14	0.0193 (11)	0.0175 (10)	0.0215 (11)	−0.0003 (8)	−0.0061 (9)	−0.0036 (9)
C15	0.0201 (12)	0.0277 (12)	0.0310 (13)	−0.0036 (9)	−0.0091 (10)	−0.0057 (10)
C16	0.0286 (13)	0.0313 (13)	0.0296 (13)	−0.0023 (10)	−0.0151 (10)	−0.0091 (10)
C17	0.0314 (13)	0.0300 (13)	0.0208 (11)	−0.0006 (10)	−0.0084 (10)	−0.0077 (10)
C18	0.0228 (12)	0.0262 (12)	0.0209 (11)	−0.0028 (9)	−0.0043 (9)	−0.0042 (9)
C19	0.0192 (11)	0.0173 (10)	0.0223 (11)	−0.0014 (8)	−0.0079 (9)	−0.0025 (9)
C20	0.0349 (13)	0.0292 (12)	0.0163 (11)	−0.0097 (10)	−0.0113 (10)	0.0006 (9)
C21	0.0169 (11)	0.0396 (14)	0.0276 (12)	−0.0076 (10)	0.0010 (9)	−0.0113 (11)
C22	0.0131 (10)	0.0177 (10)	0.0237 (11)	−0.0012 (8)	−0.0072 (8)	−0.0016 (9)
C23	0.0181 (11)	0.0242 (11)	0.0199 (11)	0.0000 (9)	−0.0055 (9)	−0.0040 (9)
C24	0.0226 (12)	0.0302 (12)	0.0197 (11)	0.0005 (9)	−0.0080 (9)	0.0002 (10)
C25	0.0280 (13)	0.0240 (12)	0.0286 (12)	0.0016 (10)	−0.0112 (10)	0.0041 (10)
C26	0.0256 (12)	0.0205 (11)	0.0296 (12)	0.0058 (9)	−0.0100 (10)	−0.0021 (10)
C27	0.0156 (10)	0.0220 (11)	0.0208 (11)	0.0008 (8)	−0.0062 (8)	−0.0030 (9)
C28	0.0151 (10)	0.0185 (10)	0.0184 (10)	−0.0001 (8)	−0.0039 (8)	−0.0025 (9)
C29	0.0170 (10)	0.0195 (11)	0.0208 (11)	−0.0010 (8)	−0.0071 (8)	−0.0044 (9)
C30	0.0236 (12)	0.0246 (12)	0.0215 (11)	0.0033 (9)	−0.0046 (9)	−0.0070 (9)
C31	0.0252 (12)	0.0345 (13)	0.0207 (11)	0.0031 (10)	−0.0019 (9)	−0.0071 (10)
C32	0.0266 (12)	0.0275 (12)	0.0177 (11)	0.0002 (10)	−0.0026 (9)	−0.0005 (9)
C33	0.0186 (11)	0.0222 (11)	0.0151 (10)	−0.0023 (9)	−0.0052 (8)	−0.0002 (9)
C34	0.0185 (11)	0.0197 (11)	0.0155 (10)	−0.0016 (8)	−0.0058 (8)	−0.0007 (8)
C35	0.0211 (11)	0.0213 (11)	0.0159 (10)	−0.0026 (9)	−0.0059 (8)	−0.0013 (9)
C36	0.0336 (13)	0.0199 (11)	0.0197 (11)	−0.0033 (9)	−0.0088 (10)	0.0018 (9)
C37	0.0393 (14)	0.0194 (11)	0.0281 (12)	0.0022 (10)	−0.0116 (11)	−0.0028 (10)
C38	0.0300 (13)	0.0258 (12)	0.0251 (12)	0.0062 (10)	−0.0062 (10)	−0.0073 (10)
C39	0.0211 (11)	0.0240 (11)	0.0165 (10)	−0.0025 (9)	−0.0041 (9)	−0.0015 (9)
C40	0.0184 (11)	0.0185 (10)	0.0162 (10)	−0.0022 (8)	−0.0070 (8)	−0.0018 (8)
C41	0.0337 (13)	0.0208 (11)	0.0269 (12)	0.0074 (10)	−0.0097 (10)	−0.0091 (10)
C42	0.0394 (15)	0.0295 (13)	0.0200 (12)	−0.0055 (11)	0.0112 (11)	0.0047 (10)
C43	0.0281 (13)	0.0408 (15)	0.0262 (13)	0.0052 (11)	−0.0049 (10)	−0.0064 (11)
C44	0.0224 (12)	0.0309 (13)	0.0259 (12)	−0.0024 (10)	−0.0031 (10)	−0.0039 (10)
C45	0.0192 (11)	0.0319 (13)	0.0261 (12)	−0.0005 (10)	−0.0041 (9)	−0.0058 (10)
C46	0.0233 (12)	0.0359 (14)	0.0226 (12)	−0.0042 (10)	−0.0040 (10)	−0.0004 (10)
C47	0.0419 (16)	0.0318 (14)	0.0295 (13)	0.0014 (11)	−0.0141 (12)	−0.0024 (11)
C48	0.0416 (16)	0.0386 (15)	0.0281 (13)	0.0126 (12)	−0.0118 (12)	−0.0103 (12)
C49	0.0267 (12)	0.0218 (11)	0.0168 (11)	0.0013 (9)	−0.0033 (9)	−0.0011 (9)
C50	0.0296 (13)	0.0258 (12)	0.0228 (12)	−0.0032 (10)	−0.0083 (10)	−0.0010 (10)
C51	0.0287 (13)	0.0239 (12)	0.0241 (12)	−0.0036 (10)	−0.0039 (10)	0.0030 (10)
C52	0.0279 (12)	0.0244 (12)	0.0177 (11)	0.0039 (10)	−0.0041 (9)	0.0051 (9)
C53	0.0243 (12)	0.0302 (12)	0.0226 (11)	0.0028 (10)	−0.0092 (9)	−0.0002 (10)
C54	0.0247 (12)	0.0238 (12)	0.0208 (11)	−0.0021 (9)	−0.0043 (9)	0.0026 (9)
C55	0.165 (6)	0.100 (4)	0.074 (3)	0.034 (4)	−0.017 (4)	0.000 (3)
C56	0.119 (4)	0.120 (4)	0.049 (2)	−0.042 (3)	−0.019 (2)	−0.020 (3)

### Geometric parameters (Å, º)

**Table:**

Zn—N8	2.1119 (17)	C14—C15	1.393 (3)
Zn—N1	2.1392 (18)	C14—C19	1.400 (3)
Zn—N10	2.1459 (18)	C15—C16	1.377 (3)
Zn—N3	2.1462 (18)	C15—H15A	0.9500
Zn—N5	2.1493 (17)	C16—C17	1.405 (3)
Zn—N6	2.2048 (18)	C16—H16A	0.9500
O1—C43	1.248 (3)	C17—C18	1.376 (3)
O2—N11	1.222 (3)	C17—H17A	0.9500
O3—N11	1.226 (3)	C18—C19	1.399 (3)
O4—N12	1.226 (3)	C18—H18A	0.9500
O5—N12	1.235 (3)	C20—H20B	0.9800
O6—N13	1.221 (4)	C20—H20A	0.9800
O7—N13	1.226 (4)	C20—H20C	0.9800
O8—C49	1.242 (3)	C21—H21A	0.9800
O9—N14	1.219 (4)	C21—H21B	0.9800
O10—N14	1.168 (4)	C21—H21C	0.9800
O11—N15	1.233 (3)	C22—C27	1.395 (3)
O12—N15	1.230 (3)	C22—C23	1.403 (3)
O13—N16	1.224 (3)	C23—C24	1.381 (3)
O14—N16	1.214 (3)	C23—H23A	0.9500
O15—C55	1.484 (7)	C24—C25	1.399 (3)
O15—H15	0.8400	C24—H24A	0.9500
O16—C56	1.417 (5)	C25—C26	1.387 (3)
O16—H16	0.8400	C25—H25A	0.9500
N1—C7	1.329 (3)	C26—C27	1.396 (3)
N1—C1	1.377 (3)	C26—H26A	0.9500
N2—C7	1.361 (3)	C28—C29	1.479 (3)
N2—C6	1.393 (3)	C29—C30	1.393 (3)
N2—C20	1.465 (3)	C30—C31	1.385 (3)
N3—C13	1.326 (3)	C30—H30A	0.9500
N3—C19	1.386 (3)	C31—C32	1.391 (3)
N4—C13	1.357 (3)	C31—H31A	0.9500
N4—C14	1.387 (3)	C32—C33	1.391 (3)
N4—C21	1.466 (3)	C32—H32A	0.9500
N5—C12	1.341 (3)	C33—C34	1.479 (3)
N5—C8	1.342 (3)	C35—C36	1.393 (3)
N6—C28	1.327 (3)	C35—C40	1.403 (3)
N6—C22	1.383 (3)	C36—C37	1.375 (3)
N7—C28	1.358 (3)	C36—H36A	0.9500
N7—C27	1.388 (3)	C37—C38	1.410 (3)
N7—C41	1.466 (3)	C37—H37A	0.9500
N8—C34	1.338 (3)	C38—C39	1.373 (3)
N8—C40	1.373 (3)	C38—H38A	0.9500
N9—C34	1.360 (3)	C39—C40	1.400 (3)
N9—C35	1.386 (3)	C39—H39A	0.9500
N9—C42	1.468 (3)	C41—H41A	0.9800
N10—C29	1.336 (3)	C41—H41B	0.9800
N10—C33	1.343 (3)	C41—H41C	0.9800
N11—C44	1.458 (3)	C42—H42A	0.9800
N12—C46	1.446 (3)	C42—H42B	0.9800
N13—C48	1.461 (4)	C42—H42C	0.9800
N14—C50	1.464 (3)	C43—C48	1.443 (4)
N15—C52	1.438 (3)	C43—C44	1.443 (4)
N16—C54	1.449 (3)	C44—C45	1.374 (3)
C1—C6	1.402 (3)	C45—C46	1.385 (4)
C1—C2	1.403 (3)	C45—H45A	0.9500
C2—C3	1.376 (3)	C46—C47	1.388 (4)
C2—H2A	0.9500	C47—C48	1.371 (4)
C3—C4	1.405 (3)	C47—H47A	0.9500
C3—H3A	0.9500	C49—C54	1.453 (3)
C4—C5	1.386 (3)	C49—C50	1.457 (3)
C4—H4A	0.9500	C50—C51	1.363 (3)
C5—C6	1.393 (3)	C51—C52	1.388 (4)
C5—H5A	0.9500	C51—H51A	0.9500
C7—C8	1.477 (3)	C52—C53	1.384 (3)
C8—C9	1.382 (3)	C53—C54	1.379 (3)
C9—C10	1.386 (3)	C53—H53A	0.9500
C9—H9A	0.9500	C55—H55A	0.9800
C10—C11	1.388 (3)	C55—H55B	0.9800
C10—H10A	0.9500	C55—H55C	0.9800
C11—C12	1.384 (3)	C56—H56A	0.9800
C11—H11A	0.9500	C56—H56B	0.9800
C12—C13	1.481 (3)	C56—H56C	0.9800
			
N8—Zn—N1	91.75 (7)	H20B—C20—H20C	109.5
N8—Zn—N10	75.08 (7)	H20A—C20—H20C	109.5
N1—Zn—N10	108.41 (7)	N4—C21—H21A	109.5
N8—Zn—N3	98.26 (7)	N4—C21—H21B	109.5
N1—Zn—N3	149.23 (7)	H21A—C21—H21B	109.5
N10—Zn—N3	102.28 (7)	N4—C21—H21C	109.5
N8—Zn—N5	115.86 (7)	H21A—C21—H21C	109.5
N1—Zn—N5	74.76 (7)	H21B—C21—H21C	109.5
N10—Zn—N5	168.82 (7)	N6—C22—C27	109.00 (19)
N3—Zn—N5	74.69 (7)	N6—C22—C23	130.6 (2)
N8—Zn—N6	148.73 (7)	C27—C22—C23	120.4 (2)
N1—Zn—N6	94.71 (7)	C24—C23—C22	117.2 (2)
N10—Zn—N6	73.85 (7)	C24—C23—H23A	121.4
N3—Zn—N6	91.64 (7)	C22—C23—H23A	121.4
N5—Zn—N6	95.34 (6)	C23—C24—C25	121.9 (2)
C55—O15—H15	109.5	C23—C24—H24A	119.0
C56—O16—H16	109.5	C25—C24—H24A	119.0
C7—N1—C1	106.06 (18)	C26—C25—C24	121.5 (2)
C7—N1—Zn	114.61 (14)	C26—C25—H25A	119.3
C1—N1—Zn	137.37 (14)	C24—C25—H25A	119.3
C7—N2—C6	106.22 (17)	C25—C26—C27	116.5 (2)
C7—N2—C20	129.15 (19)	C25—C26—H26A	121.8
C6—N2—C20	124.59 (18)	C27—C26—H26A	121.8
C13—N3—C19	105.66 (18)	N7—C27—C22	106.33 (18)
C13—N3—Zn	115.66 (14)	N7—C27—C26	131.3 (2)
C19—N3—Zn	138.31 (14)	C22—C27—C26	122.4 (2)
C13—N4—C14	106.48 (17)	N6—C28—N7	112.74 (18)
C13—N4—C21	129.72 (19)	N6—C28—C29	118.63 (19)
C14—N4—C21	123.71 (19)	N7—C28—C29	128.61 (19)
C12—N5—C8	120.67 (18)	N10—C29—C30	121.0 (2)
C12—N5—Zn	119.28 (14)	N10—C29—C28	111.03 (18)
C8—N5—Zn	118.88 (14)	C30—C29—C28	127.8 (2)
C28—N6—C22	105.66 (18)	C31—C30—C29	117.9 (2)
C28—N6—Zn	112.19 (14)	C31—C30—H30A	121.0
C22—N6—Zn	137.15 (14)	C29—C30—H30A	121.0
C28—N7—C27	106.23 (18)	C30—C31—C32	120.9 (2)
C28—N7—C41	129.40 (19)	C30—C31—H31A	119.5
C27—N7—C41	124.34 (18)	C32—C31—H31A	119.5
C34—N8—C40	106.14 (17)	C33—C32—C31	117.9 (2)
C34—N8—Zn	114.64 (14)	C33—C32—H32A	121.1
C40—N8—Zn	134.92 (14)	C31—C32—H32A	121.1
C34—N9—C35	106.88 (17)	N10—C33—C32	120.8 (2)
C34—N9—C42	129.5 (2)	N10—C33—C34	110.28 (18)
C35—N9—C42	123.61 (19)	C32—C33—C34	128.9 (2)
C29—N10—C33	121.41 (19)	N8—C34—N9	111.88 (19)
C29—N10—Zn	119.74 (14)	N8—C34—C33	118.54 (18)
C33—N10—Zn	118.83 (14)	N9—C34—C33	129.55 (19)
O2—N11—O3	123.2 (2)	N9—C35—C36	131.6 (2)
O2—N11—C44	118.6 (2)	N9—C35—C40	105.95 (18)
O3—N11—C44	118.2 (2)	C36—C35—C40	122.4 (2)
O4—N12—O5	123.0 (2)	C37—C36—C35	116.0 (2)
O4—N12—C46	118.8 (2)	C37—C36—H36A	122.0
O5—N12—C46	118.2 (2)	C35—C36—H36A	122.0
O6—N13—O7	124.2 (3)	C36—C37—C38	122.4 (2)
O6—N13—C48	116.9 (3)	C36—C37—H37A	118.8
O7—N13—C48	118.9 (3)	C38—C37—H37A	118.8
O10—N14—O9	123.3 (3)	C39—C38—C37	121.4 (2)
O10—N14—C50	119.3 (3)	C39—C38—H38A	119.3
O9—N14—C50	117.3 (3)	C37—C38—H38A	119.3
O12—N15—O11	122.6 (2)	C38—C39—C40	117.2 (2)
O12—N15—C52	119.1 (2)	C38—C39—H39A	121.4
O11—N15—C52	118.2 (2)	C40—C39—H39A	121.4
O14—N16—O13	121.9 (2)	N8—C40—C39	130.28 (19)
O14—N16—C54	118.5 (2)	N8—C40—C35	109.16 (19)
O13—N16—C54	119.5 (2)	C39—C40—C35	120.6 (2)
N1—C1—C6	109.04 (18)	N7—C41—H41A	109.5
N1—C1—C2	130.7 (2)	N7—C41—H41B	109.5
C6—C1—C2	120.3 (2)	H41A—C41—H41B	109.5
C3—C2—C1	117.4 (2)	N7—C41—H41C	109.5
C3—C2—H2A	121.3	H41A—C41—H41C	109.5
C1—C2—H2A	121.3	H41B—C41—H41C	109.5
C2—C3—C4	121.7 (2)	N9—C42—H42A	109.5
C2—C3—H3A	119.1	N9—C42—H42B	109.5
C4—C3—H3A	119.1	H42A—C42—H42B	109.5
C5—C4—C3	121.8 (2)	N9—C42—H42C	109.5
C5—C4—H4A	119.1	H42A—C42—H42C	109.5
C3—C4—H4A	119.1	H42B—C42—H42C	109.5
C4—C5—C6	116.2 (2)	O1—C43—C48	124.1 (3)
C4—C5—H5A	121.9	O1—C43—C44	124.5 (2)
C6—C5—H5A	121.9	C48—C43—C44	111.3 (2)
N2—C6—C5	131.3 (2)	C45—C44—C43	124.7 (2)
N2—C6—C1	106.09 (18)	C45—C44—N11	116.8 (2)
C5—C6—C1	122.6 (2)	C43—C44—N11	118.5 (2)
N1—C7—N2	112.57 (18)	C44—C45—C46	119.2 (2)
N1—C7—C8	119.42 (19)	C44—C45—H45A	120.4
N2—C7—C8	127.94 (19)	C46—C45—H45A	120.4
N5—C8—C9	121.4 (2)	C45—C46—C47	120.8 (2)
N5—C8—C7	110.57 (18)	C45—C46—N12	118.9 (2)
C9—C8—C7	128.0 (2)	C47—C46—N12	120.2 (2)
C8—C9—C10	117.9 (2)	C48—C47—C46	118.9 (2)
C8—C9—H9A	121.1	C48—C47—H47A	120.6
C10—C9—H9A	121.1	C46—C47—H47A	120.6
C9—C10—C11	120.9 (2)	C47—C48—C43	125.1 (2)
C9—C10—H10A	119.6	C47—C48—N13	117.3 (2)
C11—C10—H10A	119.6	C43—C48—N13	117.7 (2)
C12—C11—C10	117.8 (2)	O8—C49—C54	126.8 (2)
C12—C11—H11A	121.1	O8—C49—C50	122.0 (2)
C10—C11—H11A	121.1	C54—C49—C50	111.14 (19)
N5—C12—C11	121.4 (2)	C51—C50—C49	125.9 (2)
N5—C12—C13	110.87 (18)	C51—C50—N14	118.1 (2)
C11—C12—C13	127.7 (2)	C49—C50—N14	116.0 (2)
N3—C13—N4	112.81 (19)	C50—C51—C52	118.0 (2)
N3—C13—C12	118.74 (19)	C50—C51—H51A	121.0
N4—C13—C12	128.44 (19)	C52—C51—H51A	121.0
N4—C14—C15	131.5 (2)	C53—C52—C51	121.6 (2)
N4—C14—C19	106.15 (19)	C53—C52—N15	118.8 (2)
C15—C14—C19	122.4 (2)	C51—C52—N15	119.5 (2)
C16—C15—C14	116.4 (2)	C54—C53—C52	119.7 (2)
C16—C15—H15A	121.8	C54—C53—H53A	120.2
C14—C15—H15A	121.8	C52—C53—H53A	120.2
C15—C16—C17	121.9 (2)	C53—C54—N16	116.1 (2)
C15—C16—H16A	119.1	C53—C54—C49	123.6 (2)
C17—C16—H16A	119.1	N16—C54—C49	120.3 (2)
C18—C17—C16	121.7 (2)	O15—C55—H55A	109.5
C18—C17—H17A	119.2	O15—C55—H55B	109.5
C16—C17—H17A	119.2	H55A—C55—H55B	109.5
C17—C18—C19	117.3 (2)	O15—C55—H55C	109.5
C17—C18—H18A	121.4	H55A—C55—H55C	109.5
C19—C18—H18A	121.4	H55B—C55—H55C	109.5
N3—C19—C18	130.7 (2)	O16—C56—H56A	109.5
N3—C19—C14	108.90 (19)	O16—C56—H56B	109.5
C18—C19—C14	120.4 (2)	H56A—C56—H56B	109.5
N2—C20—H20B	109.5	O16—C56—H56C	109.5
N2—C20—H20A	109.5	H56A—C56—H56C	109.5
H20B—C20—H20A	109.5	H56B—C56—H56C	109.5
N2—C20—H20C	109.5		
			
N8—Zn—N1—C7	−127.81 (15)	N4—C14—C19—C18	179.8 (2)
N10—Zn—N1—C7	157.34 (14)	C15—C14—C19—C18	0.3 (3)
N3—Zn—N1—C7	−18.4 (2)	C28—N6—C22—C27	1.8 (2)
N5—Zn—N1—C7	−11.46 (14)	Zn—N6—C22—C27	−149.76 (17)
N6—Zn—N1—C7	82.81 (15)	C28—N6—C22—C23	−176.4 (2)
N8—Zn—N1—C1	70.9 (2)	Zn—N6—C22—C23	32.0 (4)
N10—Zn—N1—C1	−3.9 (2)	N6—C22—C23—C24	178.7 (2)
N3—Zn—N1—C1	−179.68 (17)	C27—C22—C23—C24	0.7 (3)
N5—Zn—N1—C1	−172.7 (2)	C22—C23—C24—C25	1.3 (3)
N6—Zn—N1—C1	−78.5 (2)	C23—C24—C25—C26	−1.7 (4)
N8—Zn—N3—C13	113.86 (15)	C24—C25—C26—C27	−0.2 (4)
N1—Zn—N3—C13	6.2 (2)	C28—N7—C27—C22	−0.1 (2)
N10—Zn—N3—C13	−169.71 (15)	C41—N7—C27—C22	178.5 (2)
N5—Zn—N3—C13	−0.80 (15)	C28—N7—C27—C26	179.9 (2)
N6—Zn—N3—C13	−95.89 (15)	C41—N7—C27—C26	−1.5 (4)
N8—Zn—N3—C19	−58.0 (2)	N6—C22—C27—N7	−1.0 (2)
N1—Zn—N3—C19	−165.68 (19)	C23—C22—C27—N7	177.38 (19)
N10—Zn—N3—C19	18.4 (2)	N6—C22—C27—C26	179.0 (2)
N5—Zn—N3—C19	−172.6 (2)	C23—C22—C27—C26	−2.6 (3)
N6—Zn—N3—C19	92.3 (2)	C25—C26—C27—N7	−177.7 (2)
N8—Zn—N5—C12	−96.80 (16)	C25—C26—C27—C22	2.3 (3)
N1—Zn—N5—C12	178.76 (17)	C22—N6—C28—N7	−1.9 (2)
N10—Zn—N5—C12	70.7 (4)	Zn—N6—C28—N7	157.61 (14)
N3—Zn—N5—C12	−4.93 (15)	C22—N6—C28—C29	176.65 (18)
N6—Zn—N5—C12	85.32 (16)	Zn—N6—C28—C29	−23.8 (2)
N8—Zn—N5—C8	95.50 (16)	C27—N7—C28—N6	1.3 (2)
N1—Zn—N5—C8	11.06 (15)	C41—N7—C28—N6	−177.2 (2)
N10—Zn—N5—C8	−97.0 (4)	C27—N7—C28—C29	−177.1 (2)
N3—Zn—N5—C8	−172.62 (17)	C41—N7—C28—C29	4.4 (4)
N6—Zn—N5—C8	−82.38 (16)	C33—N10—C29—C30	0.1 (3)
N8—Zn—N6—C28	24.7 (2)	Zn—N10—C29—C30	178.89 (16)
N1—Zn—N6—C28	125.93 (15)	C33—N10—C29—C28	−175.87 (19)
N10—Zn—N6—C28	18.11 (14)	Zn—N10—C29—C28	2.9 (2)
N3—Zn—N6—C28	−84.20 (15)	N6—C28—C29—N10	14.6 (3)
N5—Zn—N6—C28	−158.97 (15)	N7—C28—C29—N10	−167.1 (2)
N8—Zn—N6—C22	174.99 (18)	N6—C28—C29—C30	−161.0 (2)
N1—Zn—N6—C22	−83.8 (2)	N7—C28—C29—C30	17.3 (4)
N10—Zn—N6—C22	168.4 (2)	N10—C29—C30—C31	−0.5 (3)
N3—Zn—N6—C22	66.1 (2)	C28—C29—C30—C31	174.7 (2)
N5—Zn—N6—C22	−8.7 (2)	C29—C30—C31—C32	0.2 (4)
N1—Zn—N8—C34	−94.50 (15)	C30—C31—C32—C33	0.5 (4)
N10—Zn—N8—C34	14.09 (15)	C29—N10—C33—C32	0.6 (3)
N3—Zn—N8—C34	114.68 (15)	Zn—N10—C33—C32	−178.19 (17)
N5—Zn—N8—C34	−168.39 (14)	C29—N10—C33—C34	−178.70 (19)
N6—Zn—N8—C34	7.5 (2)	Zn—N10—C33—C34	2.5 (2)
N1—Zn—N8—C40	58.3 (2)	C31—C32—C33—N10	−0.9 (3)
N10—Zn—N8—C40	166.9 (2)	C31—C32—C33—C34	178.3 (2)
N3—Zn—N8—C40	−92.5 (2)	C40—N8—C34—N9	−0.4 (2)
N5—Zn—N8—C40	−15.6 (2)	Zn—N8—C34—N9	159.95 (14)
N6—Zn—N8—C40	160.33 (17)	C40—N8—C34—C33	−178.56 (18)
N8—Zn—N10—C29	172.27 (17)	Zn—N8—C34—C33	−18.3 (2)
N1—Zn—N10—C29	−100.90 (16)	C35—N9—C34—N8	0.5 (2)
N3—Zn—N10—C29	76.87 (16)	C42—N9—C34—N8	−177.9 (2)
N5—Zn—N10—C29	3.9 (4)	C35—N9—C34—C33	178.4 (2)
N6—Zn—N10—C29	−11.26 (15)	C42—N9—C34—C33	0.1 (4)
N8—Zn—N10—C33	−8.94 (15)	N10—C33—C34—N8	10.4 (3)
N1—Zn—N10—C33	77.89 (16)	C32—C33—C34—N8	−168.8 (2)
N3—Zn—N10—C33	−104.34 (16)	N10—C33—C34—N9	−167.4 (2)
N5—Zn—N10—C33	−177.3 (3)	C32—C33—C34—N9	13.3 (4)
N6—Zn—N10—C33	167.52 (17)	C34—N9—C35—C36	−179.9 (2)
C7—N1—C1—C6	−1.4 (2)	C42—N9—C35—C36	−1.4 (4)
Zn—N1—C1—C6	160.87 (16)	C34—N9—C35—C40	−0.4 (2)
C7—N1—C1—C2	180.0 (2)	C42—N9—C35—C40	178.1 (2)
Zn—N1—C1—C2	−17.7 (4)	N9—C35—C36—C37	179.6 (2)
N1—C1—C2—C3	176.5 (2)	C40—C35—C36—C37	0.2 (3)
C6—C1—C2—C3	−1.9 (3)	C35—C36—C37—C38	−0.2 (4)
C1—C2—C3—C4	0.8 (3)	C36—C37—C38—C39	−0.1 (4)
C2—C3—C4—C5	0.6 (4)	C37—C38—C39—C40	0.5 (4)
C3—C4—C5—C6	−0.8 (3)	C34—N8—C40—C39	179.6 (2)
C7—N2—C6—C5	177.5 (2)	Zn—N8—C40—C39	25.3 (4)
C20—N2—C6—C5	−0.5 (4)	C34—N8—C40—C35	0.1 (2)
C7—N2—C6—C1	−0.5 (2)	Zn—N8—C40—C35	−154.28 (16)
C20—N2—C6—C1	−178.50 (19)	C38—C39—C40—N8	180.0 (2)
C4—C5—C6—N2	−178.1 (2)	C38—C39—C40—C35	−0.5 (3)
C4—C5—C6—C1	−0.3 (3)	N9—C35—C40—N8	0.2 (2)
N1—C1—C6—N2	1.2 (2)	C36—C35—C40—N8	179.7 (2)
C2—C1—C6—N2	179.96 (19)	N9—C35—C40—C39	−179.41 (19)
N1—C1—C6—C5	−177.0 (2)	C36—C35—C40—C39	0.1 (3)
C2—C1—C6—C5	1.7 (3)	O1—C43—C44—C45	174.1 (3)
C1—N1—C7—N2	1.1 (2)	C48—C43—C44—C45	−2.3 (4)
Zn—N1—C7—N2	−165.78 (14)	O1—C43—C44—N11	−6.9 (4)
C1—N1—C7—C8	178.35 (18)	C48—C43—C44—N11	176.7 (2)
Zn—N1—C7—C8	11.4 (2)	O2—N11—C44—C45	143.9 (3)
C6—N2—C7—N1	−0.4 (2)	O3—N11—C44—C45	−35.2 (3)
C20—N2—C7—N1	177.5 (2)	O2—N11—C44—C43	−35.2 (4)
C6—N2—C7—C8	−177.3 (2)	O3—N11—C44—C43	145.8 (3)
C20—N2—C7—C8	0.5 (4)	C43—C44—C45—C46	2.0 (4)
C12—N5—C8—C9	1.2 (3)	N11—C44—C45—C46	−177.0 (2)
Zn—N5—C8—C9	168.72 (17)	C44—C45—C46—C47	−1.5 (4)
C12—N5—C8—C7	−175.97 (18)	C44—C45—C46—N12	175.7 (2)
Zn—N5—C8—C7	−8.4 (2)	O4—N12—C46—C45	2.0 (4)
N1—C7—C8—N5	−2.2 (3)	O5—N12—C46—C45	−176.4 (3)
N2—C7—C8—N5	174.6 (2)	O4—N12—C46—C47	179.1 (2)
N1—C7—C8—C9	−179.1 (2)	O5—N12—C46—C47	0.7 (4)
N2—C7—C8—C9	−2.4 (4)	C45—C46—C47—C48	1.4 (4)
N5—C8—C9—C10	−1.3 (3)	N12—C46—C47—C48	−175.7 (2)
C7—C8—C9—C10	175.3 (2)	C46—C47—C48—C43	−1.9 (4)
C8—C9—C10—C11	0.6 (4)	C46—C47—C48—N13	178.7 (3)
C9—C10—C11—C12	0.2 (4)	O1—C43—C48—C47	−174.2 (3)
C8—N5—C12—C11	−0.4 (3)	C44—C43—C48—C47	2.2 (4)
Zn—N5—C12—C11	−167.86 (16)	O1—C43—C48—N13	5.3 (4)
C8—N5—C12—C13	176.40 (18)	C44—C43—C48—N13	−178.4 (2)
Zn—N5—C12—C13	8.9 (2)	O6—N13—C48—C47	39.2 (4)
C10—C11—C12—N5	−0.3 (3)	O7—N13—C48—C47	−141.4 (3)
C10—C11—C12—C13	−176.5 (2)	O6—N13—C48—C43	−140.3 (3)
C19—N3—C13—N4	−0.3 (2)	O7—N13—C48—C43	39.1 (4)
Zn—N3—C13—N4	−174.72 (14)	O8—C49—C50—C51	171.5 (2)
C19—N3—C13—C12	−179.71 (18)	C54—C49—C50—C51	−5.4 (4)
Zn—N3—C13—C12	5.9 (2)	O8—C49—C50—N14	−7.1 (4)
C14—N4—C13—N3	0.4 (2)	C54—C49—C50—N14	175.9 (2)
C21—N4—C13—N3	177.0 (2)	O10—N14—C50—C51	118.4 (4)
C14—N4—C13—C12	179.7 (2)	O9—N14—C50—C51	−63.8 (4)
C21—N4—C13—C12	−3.7 (4)	O10—N14—C50—C49	−62.8 (4)
N5—C12—C13—N3	−9.7 (3)	O9—N14—C50—C49	115.0 (3)
C11—C12—C13—N3	166.9 (2)	C49—C50—C51—C52	3.8 (4)
N5—C12—C13—N4	171.1 (2)	N14—C50—C51—C52	−177.6 (2)
C11—C12—C13—N4	−12.4 (4)	C50—C51—C52—C53	−0.2 (4)
C13—N4—C14—C15	179.2 (2)	C50—C51—C52—N15	177.6 (2)
C21—N4—C14—C15	2.3 (4)	O12—N15—C52—C53	0.7 (4)
C13—N4—C14—C19	−0.3 (2)	O11—N15—C52—C53	179.3 (2)
C21—N4—C14—C19	−177.1 (2)	O12—N15—C52—C51	−177.2 (2)
N4—C14—C15—C16	−178.9 (2)	O11—N15—C52—C51	1.4 (3)
C19—C14—C15—C16	0.4 (3)	C51—C52—C53—C54	−1.0 (4)
C14—C15—C16—C17	−0.4 (4)	N15—C52—C53—C54	−178.8 (2)
C15—C16—C17—C18	−0.4 (4)	C52—C53—C54—N16	177.6 (2)
C16—C17—C18—C19	1.1 (4)	C52—C53—C54—C49	−1.3 (4)
C13—N3—C19—C18	−179.5 (2)	O14—N16—C54—C53	−159.6 (3)
Zn—N3—C19—C18	−7.2 (4)	O13—N16—C54—C53	16.6 (4)
C13—N3—C19—C14	0.2 (2)	O14—N16—C54—C49	19.3 (4)
Zn—N3—C19—C14	172.53 (16)	O13—N16—C54—C49	−164.5 (2)
C17—C18—C19—N3	178.6 (2)	O8—C49—C54—C53	−172.7 (2)
C17—C18—C19—C14	−1.1 (3)	C50—C49—C54—C53	4.1 (3)
N4—C14—C19—N3	0.1 (2)	O8—C49—C54—N16	8.5 (4)
C15—C14—C19—N3	−179.5 (2)	C50—C49—C54—N16	−174.7 (2)

### Hydrogen-bond geometry (Å, º)

**Table:**

*D*—H···*A*	*D*—H	H···*A*	*D*···*A*	*D*—H···*A*
O15—H15···O1^i^	0.84	1.94	2.756 (4)	165
C31—H31*A*···O2^i^	0.95	2.53	3.204 (3)	128
C42—H42*A*···O1^i^	0.98	2.32	3.106 (3)	137
C55—H55*C*···O9^ii^	0.98	2.56	3.415 (7)	146
C11—H11*A*···O11^iii^	0.95	2.39	3.207 (3)	144
C10—H10*A*···O3^iv^	0.95	2.37	3.233 (3)	152
C4—H4*A*···O4^v^	0.95	2.50	3.333 (3)	146
C37—H37*A*···O11^vi^	0.95	2.49	3.314 (3)	146
C10—H10*A*···O10^vii^	0.95	2.57	3.133 (4)	118
C20—H20*B*···O14^vii^	0.98	2.42	2.949 (3)	113

Symmetry codes: (i) *x*, *y*, *z*+1; (ii) *x*, *y*+1, *z*; (iii) *x*+1, *y*+1, *z*−1; (iv) *x*+1, *y*, *z*; (v) −*x*, −*y*+1, −*z*+1; (vi) −*x*+1, −*y*, −*z*+2; (vii) −*x*+1, −*y*+1, −*z*+1.
